# Environmental Health: A Global Enterprise

**DOI:** 10.1289/ehp.12802

**Published:** 2009-05

**Authors:** Hugh A. Tilson, Jane C. Schroeder

**Affiliations:** Editor-in-Chief, *EHP*, E-mail: tilsonha@niehs.nih.gov; Science Editor, *EHP*, E-mail: schroederjc@niehs.nih.gov

In the April 2008 issue of *Environmental Health Perspectives (EHP)*, Drew et al. (2008) noted that global environmental health has evolved as a high priority for the entire environmental health community. The National Institute of Environmental Health Sciences (NIEHS) has invested considerable time and resources in conjunction with other U.S.-based and international organizations to study issues relevant to global environmental health. The NIEHS 2006–2011 Strategic Plan (NIEHS 2006), for example, puts a high priority on global environmental health research, capacity-building training, and international partnerships. If “global environmental health research” is defined as research conducted outside the United States on foreign populations or environmental samples, then the NIEHS has funded 57 global environmental health research projects in 37 countries at an estimated cost of $30 million between 2005 and 2007 (Drew et al. 2008). The NIEHS has contributed in other ways to the study of global environmental health, including support for intramural researchers involved in collaborations with foreign countries, training of foreign scientists, and support for scientific conferences and meetings to build capacity in other countries (Suk 2008). The NIEHS also recently sponsored a workshop to consider potential actions and activities related to the human health effects of climate change—perhaps the quintessential global environmental health issue (Hrynkow 2008).

As noted by Suk (2008), *EHP* supports global environmental health research through its policy of open access and commitment to dissemination of research and information to the developing world. Approximately 13% of research articles published in *EHP* from 1999 through 2008 were related to global environmental health using the definition of Drew et al. (2008). Many of those papers originated in developing countries such as China, Bangladesh, and India. A recent annual update of *EHP’*s activities (Tilson 2009) described several initiatives undertaken in 2008 to restore and expand the journal’s commitment to global environmental health issues. These include renewed support for a Chinese-language edition of *EHP* published by the Shanghai Municipal Center for Disease Control and Prevention and for partnerships with other environmental and public health journals such as *Mali Médical*, *Cienca y Trabajo*, *Ciência & Saúde Coletiva*, *Salud Pública de México*, and *the Journal of Environmental and Occupational Medicine. EHP* is committed to pursuing additional partner ships with other foreign journals in 2009.

Another approach to promoting interchange and dialogue with the non-U.S. environmental health research community is to identify issues and topics of interest to this audience and make that information available to the larger international environmental health science community. To that end, *EHP* is pleased this month to publish editorials by Haidong Kan and his colleagues from Fudan University, Shanghai, China (Kan et al. 2009), and Aiguo Wang and Xuemin Chen of the Huazhong University of Science and Technology (Wang and Chen 2009). Kan et al. discuss the health impact of outdoor pollution in China, and Wang and Chen discuss the funding of basic research in China. Their editorials have been adapted from editorials originally published in Chinese. We hope these editorials will provide a window into the concerns and priorities of researchers in China.

## Figures and Tables

**Figure:**
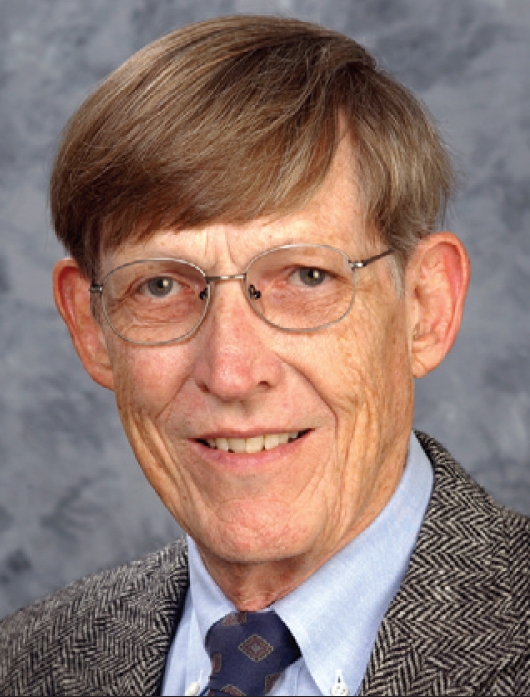
Hugh A. Tilson

**Figure:**
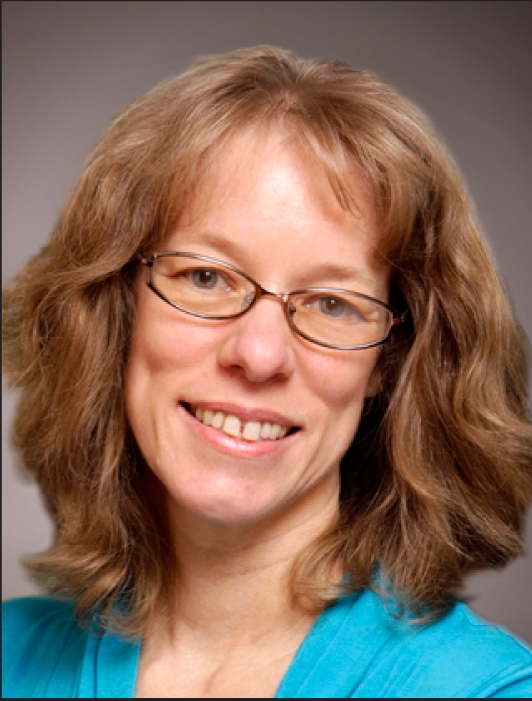
Jane C. Schroeder
